# Fate and Distribution of Heavy Metals in Wastewater Irrigated Calcareous Soils

**DOI:** 10.1155/2014/865934

**Published:** 2014-03-02

**Authors:** Mohammed Hashem Stietiya, Mohammad Duqqah, Theophilus Udeigwe, Ruba Zubi, Tarek Ammari

**Affiliations:** ^1^Department of Land, Water and Environment, Faculty of Agriculture, The University of Jordan, Amman 11942, Jordan; ^2^School of Plant and Soil Science, Texas Tech University, Lubbock, TX 79409-2122, USA; ^3^Department of Water Resources and Environmental Management, Faculty of Agricultural Technology, Al-Balqa' Applied University, Al-Salt 19117, Jordan

## Abstract

Accumulation of heavy metals in Jordanian soils irrigated with treated wastewater threatens agricultural sustainability. This study was carried out to investigate the environmental fate of Zn, Ni, and Cd in calcareous soils irrigated with treated wastewater and to elucidate the impact of hydrous ferric oxide (HFO) amendment on metal redistribution among soil fractions. Results showed that sorption capacity for Zarqa River (ZR1) soil was higher than Wadi Dhuleil (WD1) soil for all metals. The order of sorption affinity for WD1 was in the decreasing order of Ni > Zn > Cd, consistent with electrostatic attraction and indication of weak association with soil constituents. Following metal addition, Zn and Ni were distributed among the carbonate and Fe/Mn oxide fractions, while Cd was distributed among the exchangeable and carbonate fractions in both soils. Amending soils with 3% HFO did not increase the concentration of metals associated with the Fe/Mn oxide fraction or impact metal redistribution. The study suggests that carbonates control the mobility and bioavailability of Zn, Ni, and Cd in these calcareous soils, even in presence of a strong adsorbent such as HFO. Thus, it can be inferred that in situ heavy metal remediation of these highly calcareous soils using iron oxide compounds could be ineffective.

## 1. Introduction

Jordan is considered as one of the world's poorest nations in renewable water resources with a per capita share of less than 148 m^3^ annually, far below the absolute water scarcity level of 500 m^3^/capita/year [1, 2]. The abnormal growth in population in the past decades due to influx of refugees from neighboring countries has placed enormous stress on what was already a limited resource. The deficit between water supply and demand in 2007 was 565 million cubic meters (MCM) [3]. The nation's water situation instigated its Ministry of Water and Irrigation in 2009 to set a national water strategy aiming at meeting water needs and reducing pressure on the limited commodity by the year 2022 [4]. This included the promotion of nonconventional water resources such as greywater, treated wastewater (TWW), and desalination of brackish water. Treated wastewater (TWW) is the most promising nonconventional resource in the country providing more than 110 MCM of water for irrigation and comprising 10% of the total water resources [5].

The largest treatment plant in Jordan is Khribet As-Samra Waste Stabilization Ponds (KS) located 30 km north east of the capital Amman, which provides 70% of all treated wastewater generated in the country [5, 6]. Despite that usage of TWW for irrigation has provided ample water supply, its use typically comes at a cost. Salinity, specific ion toxicities, elevated concentrations of pathogens, heavy metals, and an array of organic and inorganic pollutants place restrictions on its use for irrigation [7, 8]. Although heavy metals in KS effluent were found to be low and within recommended standard limits for irrigation water, heavy metal contamination in soils irrigated from KS effluent has been reported [8–11]. Ghrefat et al. [12] reported that soils were polluted with Pb, Cd, Mn, and Cu, while Abderahman and Abu-Rukah [13] reported low to moderate pollution with Pb and Ni and slight to moderate pollution with Cu, Zn, and Cr. Heavy metal accumulation in soils is expected to increase as KS becomes inadequate to handle water quantities requiring treatment, hence discharging low quality effluent [3]. There is a need to control metal mobility in the receiving soils to prevent their transfer into the food chain or groundwater aquifer, notably since KS effluent stream is considered as the main source of recharge for Al-Sukhneh Aquifer [14].

One method of controlling metal mobility is the application of iron oxide-based amendments to the soil which may be a cost effective, “in situ” approach to restore contaminated soils and wastewaters [16, 17]. Heavy metals may form strong inner sphere complexes with the pH dependent charges of iron oxides, thus, becoming unavailable [18, 19]. Even at low pH where the mineral surface is positively charged, iron oxide mineral surfaces have the ability to specifically adsorb metals such as Zn [20]. In fact, mobility and bioavailability of metals are largely controlled by iron oxide minerals in soils high in oxide content [19, 21]. In calcareous soils, such as those investigated here, variable charge surfaces of iron oxides are expected to bear negative charges due to high pH, providing surface for positively charged metal ions to bind [20]. Hydrous ferric oxide (HFO) is an amorphous iron oxide mineral with large surface area compared to other crystalline oxides such as hematite or goethite [22]. The addition of large surface area HFO to soils irrigated with treated wastewater from KS may play a role in reducing the mobility of Zn, Ni, and Cd in these soils.

Although heavy metal content in soils irrigated with KS wastewater has been reported, no study so far has investigated heavy metal sorption characteristics, fractions, and environmental fate in these soils. If agriculture is to remain a viable and sustainable option in the area using KS treated wastewater, it is imperative that measures can be ensured to control metal mobility, availability, and possible transfer to the food chain. In this study, we aim at evaluating the environmental fate of Zn, Ni, and Cd in characteristic semidesert calcareous soils irrigated with treated wastewater by investigating the (1) sorption behaviors of these heavy metals and (2) distribution of these metals among soil chemical components. Findings will provide baseline information for devising effective management strategies in this region and its surroundings that are currently facing complex environmental challenges.

## 2. Materials and Methods

### 2.1. Site Description and Soil Analysis

**Figure 1:**
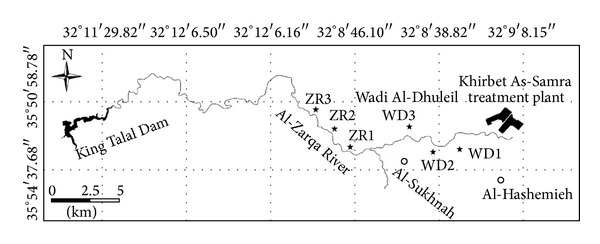
Map of the study area showing the location of investigated soils irrigated with treated wastewater from Khirbet As-Samra effluent along the Zarqa River, Jordan.

The soils in this study were collected from fields irrigated with treated wastewater (TWW) discharged from Khirbet As-Samra plant (KS) (Figure 1). The study area lies between 32°8′ and 32°10′ N latitude and between 36°10′ E and 36°0′ E longitude. The effluent is discharged from KS into Wadi Dhuleil, a tributary of Zarqa River, which then converges with the seasonally flowing river near Al-Sukhnah village. The river flows 42 km until it reaches the King Talal Dam (KTD).

On the basis of irrigation water quality, the study area can be divided into two regions: the first includes areas irrigated along Wadi Dhuleil where the water used is treated wastewater only, and the second includes areas irrigated along Zarqa River where treated wastewater is seasonally blended with surface water. Soils collected from these two zones are henceforth referred to as WD (Wadi Dhuleil) and ZR (Zarqa River) soils. Three composite samples were taken from each zone (WD and ZR) at depths of 0–20, 20–40, and 40–60 cm. Soils were air dried, ground to pass a 2 mm sieve, and stored in clean polyethylene bottles prior to analysis. Soils were analyzed for particle-size distribution using hydrometer method [23], cation exchange capacity (CEC) by saturating with ammonium acetate (NH_4_OAc) at pH 7.0 [24], total organic carbon content using the Walkley-Black method [25], calcium carbonate content by titration, exchangeable cations (Ca, Mg, K, Na, Fe) using the BaCl_2_ method [24], NO_3_^−^ and exchangeable NH_4_^+^ using automated spectrophotometer after extraction with 2.0 M KCl [26], and pH and electrical conductivity (EC) in soil paste extracts using pH and EC meters [24].

Total elemental analysis was determined using Atomic Absorption Spectroscopy (AAS) (AAnalyst 700, Perkin-Elmer Inc.,USA) after digestion with HNO_3_-HCl according to the USEPA 3050-B method [27]. Extracts were analyzed for Zn, Cu, Ni, Cd, Cr, and Pb. Content of free Fe oxide was determined by citrate-bicarbonate-dithionate method [28]. Hydrous ferric oxide (HFO) used in this study was synthesized according to the procedure described by Schwertmann and Cornell [22]. Briefly, 0.1 M of Fe(NO_3_)_2_ was neutralized with 1 M NaOH. The produced HFO was washed several times with deionized water to remove excess salt. The electrical conductivity of the water was continuously monitored and water was changed several times a day until salt free. The BET-surface area of the synthesized HFO was expected to be within 200–320 m^2 ^g^−1^ [22]. All reagents used in the present investigation were of analytical reagent grade.

### 2.2. Sorption Isotherms

Sorption isotherms of Zn, Ni, and Cd were constructed for WD1 and ZR1 soils, each representing a distinct area in terms of irrigation water quality. Isotherms were obtained by adding 30 mL of varying concentrations of metal solution to 50 mL centrifuge tubes containing 0.5 g of soil. Solutions of Zn, Ni, and Cd were prepared from salts of Zn(NO_3_)_2_, Cd(NO_3_)_2_, and Ni(NO_3_)_2_, respectively. Initial concentrations (*C*_*i*_) of Cd, Zn, and Ni were 0.0, 2.0, 4.0, 8.0, 12.0, 20.0, 30.0, 40.0, and 50.0 mg L^−1^. Tubes were placed on a reciprocating shaker for 1 week to reach equilibration, centrifuged, filtered to separate solution from soil phase, and supernatant was then acidified using 0.1 M HNO_3_. Equilibrium concentrations (*C*_*e*_) of Zn, Ni, and Cd were determined using AAS and sorbed concentrations (*q*) were calculated as the difference between *C*_*i*_ and *C*_*e*_ expressed as follows:

(1) $$\begin{array}{c} q=\frac{\left(C_{i}-C_{e}\right)*v}{\text{wt}}, \end{array}$$

where *q* is sorbed quantity in mg kg^−1^; *v* is volume of metal solution in L; wt is weight of soil in kg. The experimental data was then fit to the Langmuir and Freundlich models using nonlinear regression [29]. The nonlinear Langmuir model is expressed as

(2) $$\begin{array}{c} q=\frac{q_{\max}K_{L}C_{e}}{1+K_{L}C_{e}}, \end{array}$$

where *q* is amount of Zn or Cd adsorbed per unit weight of soil (mg kg^−1^), *C*_*e*_ is the equilibrium concentration (mg L^−1^), *q*_max⁡_ is the monolayer sorption capacity (mg kg^−1^), and *K*_*L*_ is the constant related to the free energy of sorption (L mg^−1^). The Freundlich model is expressed as

(3) $$\begin{array}{c} q_{e}=K_{d}C_{e}^{N}, \end{array}$$

where *K*_*d*_ is the constant indicative of the relative sorption capacity of soil (mg kg^−1^), *N* is the constant indicative of the intensity of sorption, and *C*_*e*_ was previously defined.

### 2.3. Fractionation of Zn, Ni, and Cd

The distribution of Zn, Ni, and Cd among soil fractions was investigated in WD1 and ZR1 soils at two initial metal concentrations (1280 and 3200 mg kg^−1^) and in presence or absence of 3% HFO. Initially, 1.0 g of soil was weighed in 50 mL centrifuge tubes and a portion of the tubes was amended with 3% (w/w) HFO while another remained nonamended. Then, 32 mL of 40 mg L^−1^ or 100 mg L^−1^ Zn, Cd, or Ni solution was added to the tubes giving 1280 and 3200 mg kg^−1^, respectively. Tubes were shaken for 1 week until equilibration was reached, centrifuged to separate the liquid from the solid phase, and filtered, and the supernatant was acidified using 0.1 M HNO_3_. Equilibrium concentrations (*C*_*e*_) of Zn, Ni, and Cd were determined using Atomic Absorption Spectroscopy (AAS). Sorbed concentrations (*q*) of Cd, Zn, and Ni were calculated as explained in (1). Successive extractions were performed on the same centrifuge tubes to determine the metal distribution in the two selected soils using the procedure developed by Tessier et al. [30]. The five fractions included the soluble and exchangeable fraction (F1) extracted using 1 M MgCl_2_; carbonate-bound fraction (F2) extracted using 1 M NaOAc at pH 5.0; easily reducible Mn and Fe oxide fraction (F3) extracted using dithionite-citrate-bicarbonate [28]; organic bound fraction (F4) extracted using H_2_O_2_ and 0.02 M HNO_3_; and residual fraction (F5) extracted by digestion using USEPA method 3050-B [27]. Equilibrium metal concentrations were determined using Atomic Absorption Spectroscopy (AAS). Standards for all trace metals analyzed were in the same matrix as the extractant to minimize extractant effects. The percent recovery of metals was calculated by dividing the sum of sequential extraction steps for each metal (*n*) by the total added concentration of metal after correcting for the control as follows:

(4) $$\begin{array}{c} \text{Recovery}\;\left(\%\right)=\left(\frac{\sum_{n}\text{sequential}\;\text{extraction}\;\text{steps}}{\text{total}\;\text{metal}\;\text{concentration}}\right)\times 100. \end{array}$$

### 2.4. Statistical Analysis

Analysis of variance statistical analysis was performed using SPSS 17 (SPSS, Inc. Chicago, IL) and comparison of means was undertaken using *t*-test to determine any significant differences at *P* ≤ 0.05.

## 3. Results and Discussion

### 3.1. Soil Characteristics

Table 1 shows the chemical and physical properties of Wadi Dhuleil (WD) and Zarqa River (ZR) soils. Soils are generally medium textured, moderately alkaline, with moderate CEC, low to moderate organic matter (OM) content, marginally saline for WD2 and ZR1 soils, and marginally sodic for WD1, WD2, and ZR1 soils [31]. The above typical OM content of these arid soils was likely due to the organic load of treated wastewater, soil amendment with manure, and continuous cultivation. The fact that some of the soils (WD1, WD2, ZR1) were marginally saline or sodic highlights the impact of irrigating with treated wastewater and other agricultural practices on soil salinization and sodication. Variation in salinity may be explained by the fact that many farmers use water excessively beyond crop water requirements, unintentionally accounting for salt leaching beyond the shallow root zone. Further studies are required to investigate the impact of agricultural practices (irrigation, tillage, fertilization, etc.) on salt buildup and structural deterioration of these soils.

Table 2 shows the total content of Zn, Cd, Ni, Cu, and Pb in soils at three depths. Obviously, there was little variation in metal concentration both spatially and with depth for the same element. This indicated that despite seasonal variation in irrigation water quality between WD and ZR soils, the accumulation of heavy metals in the soils did not vary likely because water quality changes only during limited periods of the winter semester in addition to the common practice of excessive leaching. The concentration of metals reported here was within the variable range of values reported by [12] and generally borderline to the critical soil concentrations for Zn and Cd as defined by Kabata-Pendias and Pendias [15]. Since Cd is a toxic metal with no apparent biological function, accumulation in these soils may limit their agricultural use.

### 3.2. Metal Sorption Characteristics and Environmental Fate Implications

**Figure 2:**
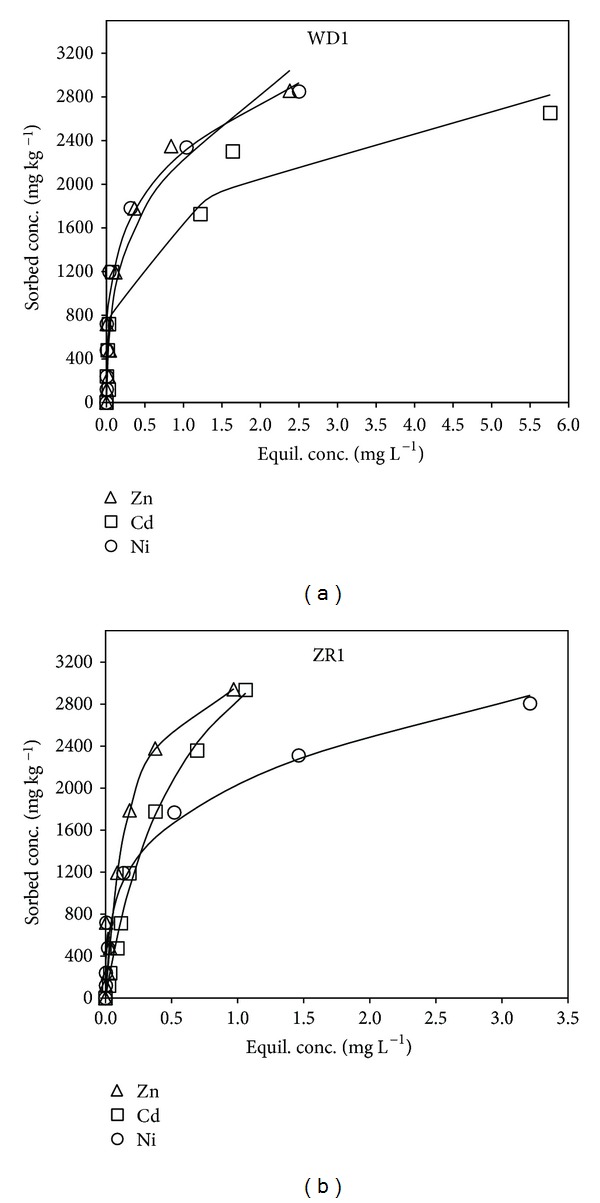
Adsorption isotherms of Zn, Cd, and Ni in WD (a) and ZR (b) soils. The solid lines represent modeled adsorption data.

Figure 2 shows the sorption isotherms of Zn, Ni, and Cd in WD1 and ZR1 soils. Because of the similarity in chemical and physical properties among the WD soils as well as among the ZR soils as evidenced from Tables 1 and 2, WD1 and ZR1 were chosen as representative units for sorption and fractionation investigations. At low equilibrium concentrations, Zn, Ni, and Cd exhibited high sorption affinity to both soils. At higher equilibrium concentrations, variation in metal sorption behavior in soils became evident. WD1 soil exhibited higher affinity for Zn and Ni than for Cd, whereas ZR1 soil exhibited higher affinity for Zn and Cd than for Ni (Figure 2).

Equilibrium concentrations of Zn, Ni, and Cd produced satisfactory fits to both Freundlich and Langmuir models as indicated by *R* ^2^ values (Table 3). Zinc and Cd sorption data were similarly fitted to both models, while Ni was slightly better fitted to the Freundlich model (average *R* ^2^ = 0.97) compared to Langmuir model (average *R* ^2^ = 0.92) in both soils. Sorption of metals was favorable in both soils as indicated by *N* values of the Freundlich model which reflect intensity of sorption and where values ranging from 0 to 1 represent favorable sorption [32, 33]. The sorption capacity, *q*_max⁡_, for ZR1 soil was higher than WD1 for all metals indicating higher sorption affinity of the former soil (Table 3). The largest difference in *q*_max⁡_ was for Cd with values of 2370.7 mg kg^−1^ and 4580.9 mg kg^−1^ in WD1 and ZR1 soils, respectively. The distribution coefficient and Freundlich constant, *K*_*d*_, values are indicators of sorption strength, where higher values indicate stronger metal sorption to soil surfaces and lower metal solubility [32]. The order of sorption affinity for WD1 and ZR1 soils according to *K*_*d*_ was in the decreasing order of Ni > Zn > Cd and Zn > Cd > Ni, respectively. The order of sorption for WD1 soil was consistent with electrostatic attraction to soil surfaces, where metal ions of smaller ionic radii are more strongly and preferentially adsorbed (ionic radii of Ni = 0.069 nm, Zn = 0.074, and Cd = 0.097 nm) [32, 34]. This suggests that metals in WD1 soil are weakly bounded to soil particles and may be easily released into the environment. On the basis of chemisorption, metal ions with higher electronegativity would be preferentially adsorbed in the decreasing order of Ni > Cd > Zn [32]. The results indicate that sorption in ZR1 soil could not be explained by either electrostatic attraction or chemisorption despite the higher affinity of this soil for Zn, Ni, and Cd as previously mentioned. Similarly, Antoniadis et al. [32] reported that metal sorption in sewage sludge-amended soil followed the order of Zn > Cd > Ni, which could not be predicted by any affinity sequence model. This indicated that ZR1 soil exhibited processes other than simple sorption to soil surfaces such as surface precipitation or ternary complex formation, which would need to be verified using microscopic techniques such as extended X-ray absorption fine structure spectroscopy (EXAFS).

### 3.3. Distribution of Metal Fractions among Soil Chemical Components

**Figure 3:**
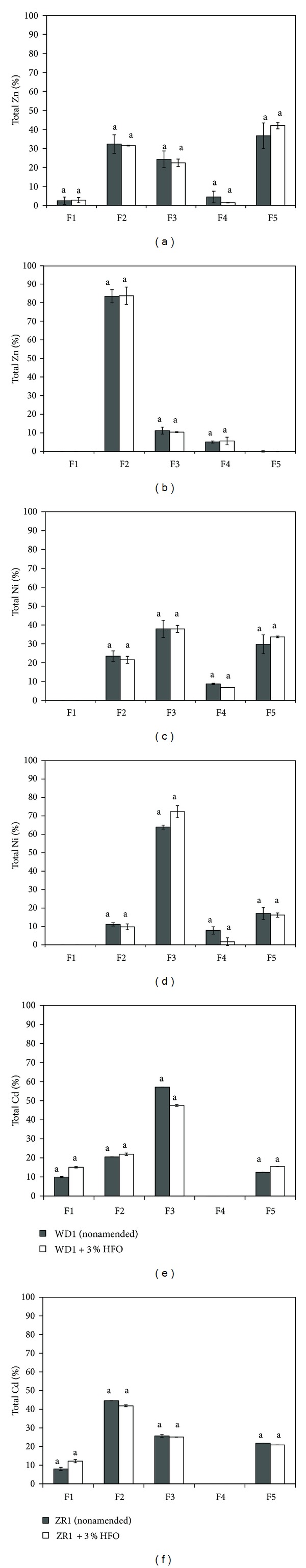
Relative distribution of Zn, Cd, and Ni in WD1 and ZR1 soils amended or nonamended with 3% hydrous ferric oxide (HFO). Mean values followed by the same letter within a column are not different at *P* < 0.05 by *t*-test. F1: soluble and exchangeable; F2: carbonate bound; F3: Fe/Mn oxide; F4: organic bound; F5: residual.

Figure 3 shows the relative distribution of Zn, Ni, and Cd within the five soil fractions in WD1 and ZR1 soils prior to metal addition. There was an obvious variation in Zn and Ni distribution in the two soils. While Zn and Ni were mainly distributed among the carbonate (F2), Fe/Mn oxide (F3), and residual fractions (F5) in WD1 soil, they were mainly associated with the carbonate and Fe/Mn oxide fractions, respectively, in ZR1 soil. In the nonamended ZR1 soil, 83.7% of total recoverable Zn was in the carbonate fraction and 64% of Ni was in the Fe/Mn oxide fraction. Cadmium was distributed among all but the OM fraction in both soils with ~10% of the total recoverable portion in exchangeable form. The mobility factor (MF) used as a relative index of metal mobility was determined based on the ratio of exchangeable and carbonate fractions to the sum of all fractions (data not shown) [35, 36]. The MF was higher for Zn and Cd compared to Ni in both soils due to higher association of the former with the carbonate and/or exchangeable fractions. Additionally, Zn and Cd were potentially more mobile in ZR1 than in WD1 soil where MF was in the decreasing order of Zn in ZR1 > Cd in ZR1 > Zn in WD1 > Cd in WD1 > Ni in WD1 > Ni in ZR1. The addition of 3% w/w hydrous ferric oxide (HFO) to soils did not impact the natural distribution of Zn, Ni, and Cd since majority of the metal pool were not present in soluble form in soil solution.

**Figure 4:**
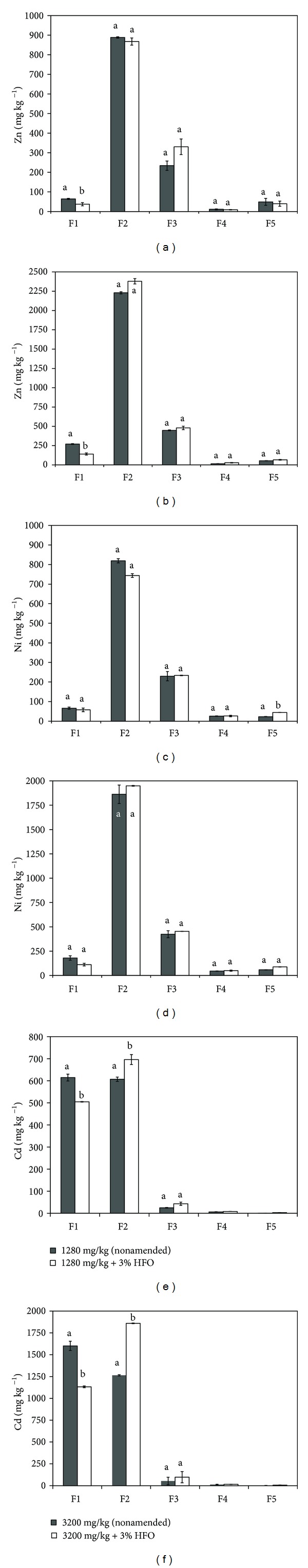
Distribution of Zn, Ni, and Cd in WD1 soil with and without HFO amendment following addition of 1280 mg kg^−1^ ((a), (c), and (e)) and 3200 mg kg^−1^ ((b), (d), and (f)) of metal solution. Mean values followed by the same letter within a column are not different at *P* < 0.05 by *t*-test. F1: soluble and exchangeable; F2: carbonate bound; F3: Fe/Mn oxide; F4: organic bound; F5: residual.

**Figure 5:**
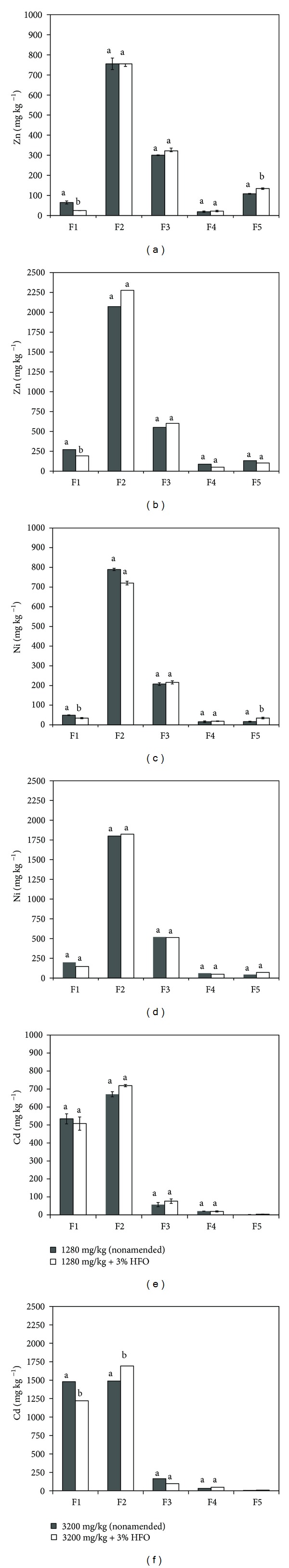
Distribution of Zn, Ni, and Cd in ZR1 soil with and without HFO amendment following addition of 1280 mg kg^−1^ ((a), (c), and (e)) and 3200 mg kg^−1^ ((b), (d), and (f)) of metal solution. Mean values followed by the same letter within a column are not different at *P* < 0.05 by *t*-test. F1: soluble and exchangeable; F2: carbonate bound; F3: Fe/Mn oxide; F4: organic bound; F5: residual.

Figures 4 and 5 show the fractionation of Zn, Ni, and Cd following metal incubation at 1280 mg kg^−1^ and 3200 mg kg^−1^ and amendment with 3% HFO in WD1 and ZR1 soils, respectively. The recovery of Zn, Ni, and Cd using the sequential extraction procedure ranged from 98.0 to 108.3, 82.9–91.8, and 97.4–106.8, respectively. The distribution of metals in both soils following metal addition was different than their native distribution. It can be seen that Zn and Ni were distributed among the carbonate and to a lesser extent the Fe/Mn oxide fractions in both soils regardless of HFO amendment, while Cd was distributed among the exchangeable and carbonate fractions with minor presence in the Fe/Mn oxide fraction. Overall, minimal concentrations of Zn, Ni, and Cd were present in the OM and residual fractions, except for residual Zn in ZR1 soil at 1280 mg kg^−1^ added concentration (Figure 4). Obviously, the carbonate was the major fraction controlling the mobility of metals in both soils following metal addition, in addition to the exchangeable fraction in the case of Cd. In the nonamended WD1 soil at 1280 mg kg^−1^ added concentration, carbonate-bound Zn, Ni, and Cd were 888.2, 819.3, and 607.0 mg kg^−1^ constituting 71.3, 70.5, and 48.4% of total recoverable concentrations, respectively (Figure 4). Incubation at 3200 mg kg^−1^ increased the concentration of metals bound to main fractions, whereas the relative distribution (%) of metals remained relatively the same. To illustrate, at the higher added concentration of 3200 mg kg^−1^, carbonate-bound Zn, Ni, and Cd in WD1 soil were 2228.7, 1862.4, and 1264.0 mg kg^−1^ constituting 73.9, 72.4, and 43.1% of total recoverable concentration, respectively (Figure 5). The same trend was found for ZR1 soil where the percentages of total recoverable carbonate-bound Zn, Ni, and Cd were 59.2, 62.4, and 53.7% at 1200 mg kg^−1^ and 66.5, 68.6, and 46.9% at 3200 mg kg^−1^ added concentrations, respectively.

The results revealed that metals were mainly associated with the mobile fraction of soils, indicating the high potential mobility and bioavailability of these metals. This is consistent with the electrostatic attraction of metals in WD1 soils as indicated earlier. The strong association of Cd with the carbonate fraction has been reported in calcareous soils, attributed to its precipitation in the form of CdCO_3_ or by replacing Ca in calcite crystals, which explains the results presented here [37–39]. Zinc and Ni on the other hand have greater tendency to become unavailable through association with the Fe/Mn oxide and residual fractions [40]. Nickel is siderophilic with high affinity for Fe oxides and less affinity for carbonates [41–45]. Yet in this study Zn and Ni were found mostly associated with the carbonate fraction following incubation. This may be due to several reasons: metals tend to be more bioavailable in light-textured soils such as in this study and in the coarse fraction of heavy soils due to low surface area and low CEC [46]. Additionally, the carbonate content of the soils was high (Table 1) allowing carbonated chemical forms to impact the reactivity and mobility of metals as had been reported for highly contaminated calcareous soils [47]. From a practical aspect, the application of lime to calcareous soils was found to decrease exchangeable Cd, Cu, Pb, and Zn and increased the carbonate bound fraction [17]. Our study highlights the importance of carbonates in retaining metals not only those with typically high carbonate affinity such as Cd but also those of affinity for nonmobile fractions such as Zn and Ni. Amending soils with 3% w/w HFO did not impact the distribution of Zn, Ni, and Cd among the soil fractions as can be seen in Figures 4 and 5. The only noticeable change following HFO amendment was the distribution of Cd between the exchangeable and carbonate fractions, where exchangeable Cd concentrations decreased and carbonate-bound Cd increased following amendment. The results showed that carbonates remained the major fraction controlling metal mobility in these Jordanian soils even after they were amended with strong adsorbent such as HFO. This is despite that iron oxide-based soil amendments such as red mud have been effectively used for the immobilization of various metals [48]. Red mud amendment was efficient in shifting Cd and Zn from the exchangeable fractions to the Fe oxide fraction [48]. However, the soils investigated in our study were of high carbonate content (Table 1) which could have overwhelmed any other soil fraction including Fe/Mn oxide fraction. It can be said that carbonates were the dominate soil fraction controlling metal retention not because of low Fe oxide content, but due to the high content of carbonate minerals which dominated the system.

## 4. Conclusion

Sorption isotherm data showed that between the 2 TWW-impacted calcareous soils (WD and ZR) examined, the ZR1 soil had higher affinity for Zn, Ni, and Cd than WD1 soil as indicated by the Langmuir sorption capacity, *q*_max⁡_. The order of sorption affinity for WD1 and ZR1 soils was in the decreasing order of Ni > Zn > Cd and Zn > Cd > Ni, respectively. This was consistent with electrostatic attraction to soil surfaces for WD1 which indicated that metals were weakly bound to soil particles, posing a potential threat of release into the environment. Addition of metals to soils resulted in distribution of Zn and Ni among the carbonate and to a lesser extent the Fe/Mn oxide fractions, while Cd was distributed among the exchangeable and carbonate fractions at both added concentrations. Amending soils with 3% HFO did not increase the concentration of metals associated with Fe/Mn oxide fraction, despite the generally high affinity of Zn and Ni for the Fe/Mn oxide fraction. The carbonate fraction was the major fraction controlling the mobility of Zn, Ni, and Cd in these soils, even when soils are amended with HFO, a strong adsorbent. This may mean that in situ remediation of these highly calcareous soils using iron oxides would be unsuccessful because carbonates will overwhelm the system.

## Figures and Tables

**Table 1:** Chemical and physical properties of Wadi Dhuleil (WD) and Zarqa River (ZR) soils at 0–20 cm depth.

Parameter	Unit	Soil sample					
		WD1	WD2	WD3	ZR1	ZR2	ZR3
Particle size		SL	SL	SL	SL	SL	SL
pH		8.4	8.3	8.4	8.3	8.6	8.2
EC	dS m^−1^	2.1	3.2	2.2	3.7	3.0	2.2
CEC	cmol kg^−1^	19.4	20.7	21.1	22.3	17.2	20.3
ESP	%	8.9	9.2	2.6	9.9	1.8	3.9
OM	%	2.0	1.1	2.1	1.6	1.0	2.1
CaCO_3_	%	33.1	32.5	36.1	40.6	61.6	22.3
Fe	%	0.56	0.63	0.58	0.51	0.51	0.53
Ca	mg kg^−1^	2759.1	2891.1	5508.8	3549.0	2421.3	5051.4
Mg	mg kg^−1^	552.6	594.3	178.8	737.1	175.5	475.2
Na	mg kg^−1^	398.0	439.7	125.6	509.2	70.0	181.2
K	mg kg^−1^	42.3	49.9	29.4	26.9	36.8	31.9
Fe	mg kg^−1^	3.6	3.6	3.5	3.7	3.9	3.5
NH_4_^+^	mg kg^−1^	18.7	37.0	18.6	14.8	31.6	43.0
NO_3_^−^	mg kg^−1^	35.0	37.0	43.5	87.2	39.6	94.8

**Table 2:** Total elemental content of WD and ZR soils at depths of 0–20, 20–40, and 40–60 cm.

Metal		Soil sample						Critical values^a^
	Depth	WD1	WD2	WD3	ZR1	ZR2	ZR3	
	cm	mg kg^−1^						
Zn	0–20	60.1	65.7	74.7	61.7	69.3	66.2	70–400
	20–40	55.9	—	71.2	63.0	67.0	62.5	
	40–60	60.1	—	—	63.1	69.7	—	

Cd	0–20	1.8	1.9	2.0	2.5	4.0	2.8	3–8
	20–40	1.7	—	2.1	2.6	3.6	3.3	
	40–60	1.6	—	—	2.4	4.3	—	

Ni	0–20	43.9	42.6	46.3	36.5	40.7	39.1	100
	20–40	41.8	—	43.7	38.5	40.5	37.2	
	40–60	45.2	—	—	46.4	40.7	—	

Cu	0–20	18.8	19.1	20.7	18.0	26.7	18.9	60–125
	20–40	17.5	—	20.7	18.6	23.1	18.3	
	40–60	18.8	—	—	18.3	23.6	—	

Pb	0–20	25.7	25.1	23.8	31.2	29.0	27.1	100–400
	20–40	23.5	—	25.5	31.1	25.2	28.3	
	40–60	23.7	—	—	30.0	28.6	—	

^a^Cited from Kabata-Pendias and Pendias, 1984 [15].

**Table 3:** Parameters of Langmuir and Freundlich isotherms for sorption of Zn, Cd, and Ni in WD1 and ZR1 soils.

Metal ion	Soil	Freundlich model			Langmuir model		
		*K*_*F*_ mg kg^−1^	*N*	*R* ^2^	*q*_max⁡_ mg kg^−1^	*K*_*L*_	*R* ^2^
Zn	WD1	2247.3	0.35	0.94	3028.8	4.61	0.94
	ZR1	3164.6	0.42	0.94	3476.9	5.72	0.95

Cd	WD1	1711.6	0.29	0.93	2370.7	10.00	0.92
	ZR1	2933.6	0.63	0.98	4580.9	1.64	0.99

Ni	WD1	2295.6	0.27	0.96	2462.2	28.40	0.91
	ZR1	2033.4	0.30	0.98	2692.0	5.89	0.93
